# Supplementary material for: Aotus nancymaae model predicts human immune response to the placental malaria vaccine candidate VAR2CSA
Source: Lab Anim (NY). 2023 Nov 6;52(12):315–23. doi: 10.1038/s41684-023-01274-2 (PMC10689237; doi:10.1038/s41684-023-01274-2)
Supplement: Supplementary file 1 — Supplementary Figs. 1–14 and Tables 1 and 2. [file 41684_2023_1274_MOESM1_ESM.pdf]

nature portfolio

https://doi.org/10.1038/s41684-023-01274-2

Supplementary information

Aotus nancymaae model predicts human immune response to the placental malaria vaccine candidate VAR2CSA

In the format provided by the authors and unedited

Supplementary Fig. 1: Flow-chart of monkeys that received monomers and conjugates of PMV

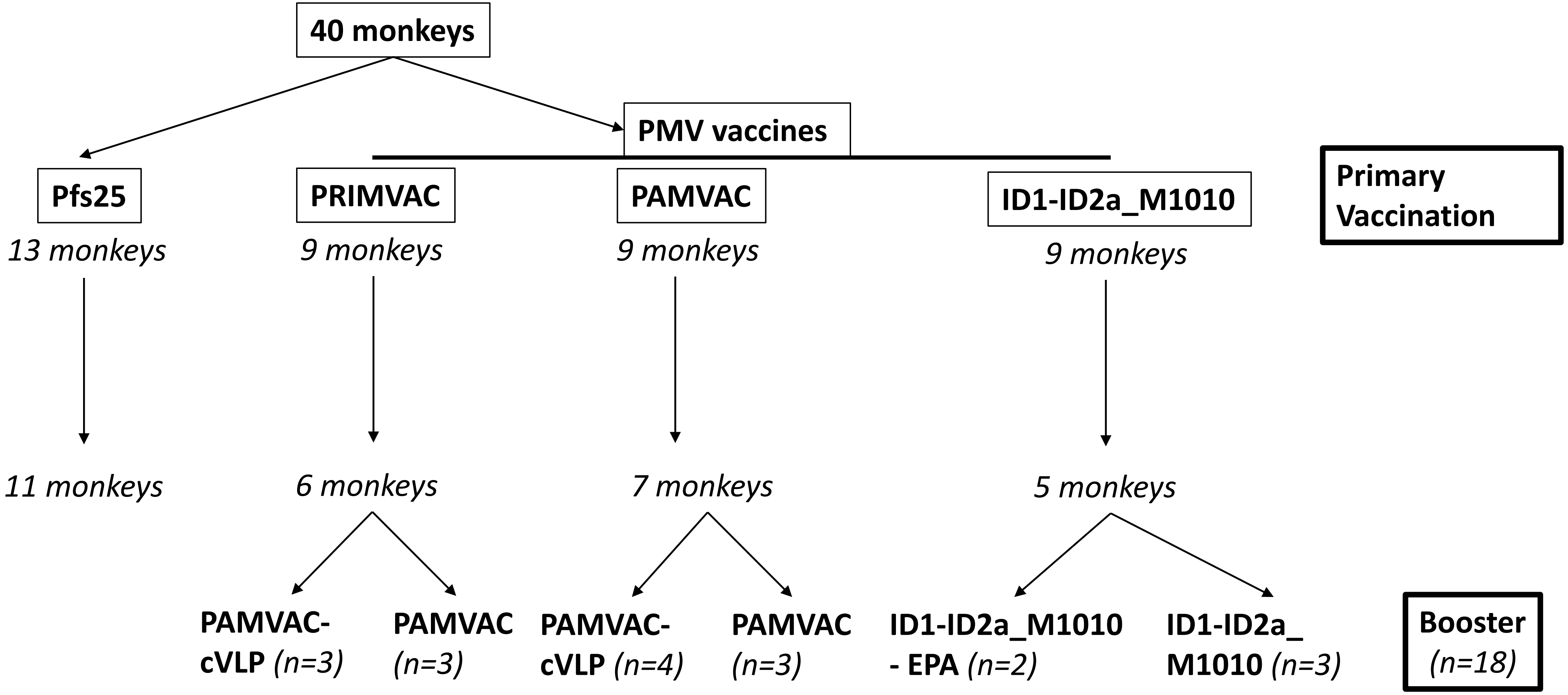

Supplementary Fig. 2: ELISA cross-reactivity of PMV-induced antibodies among VAR2CSA antigens.

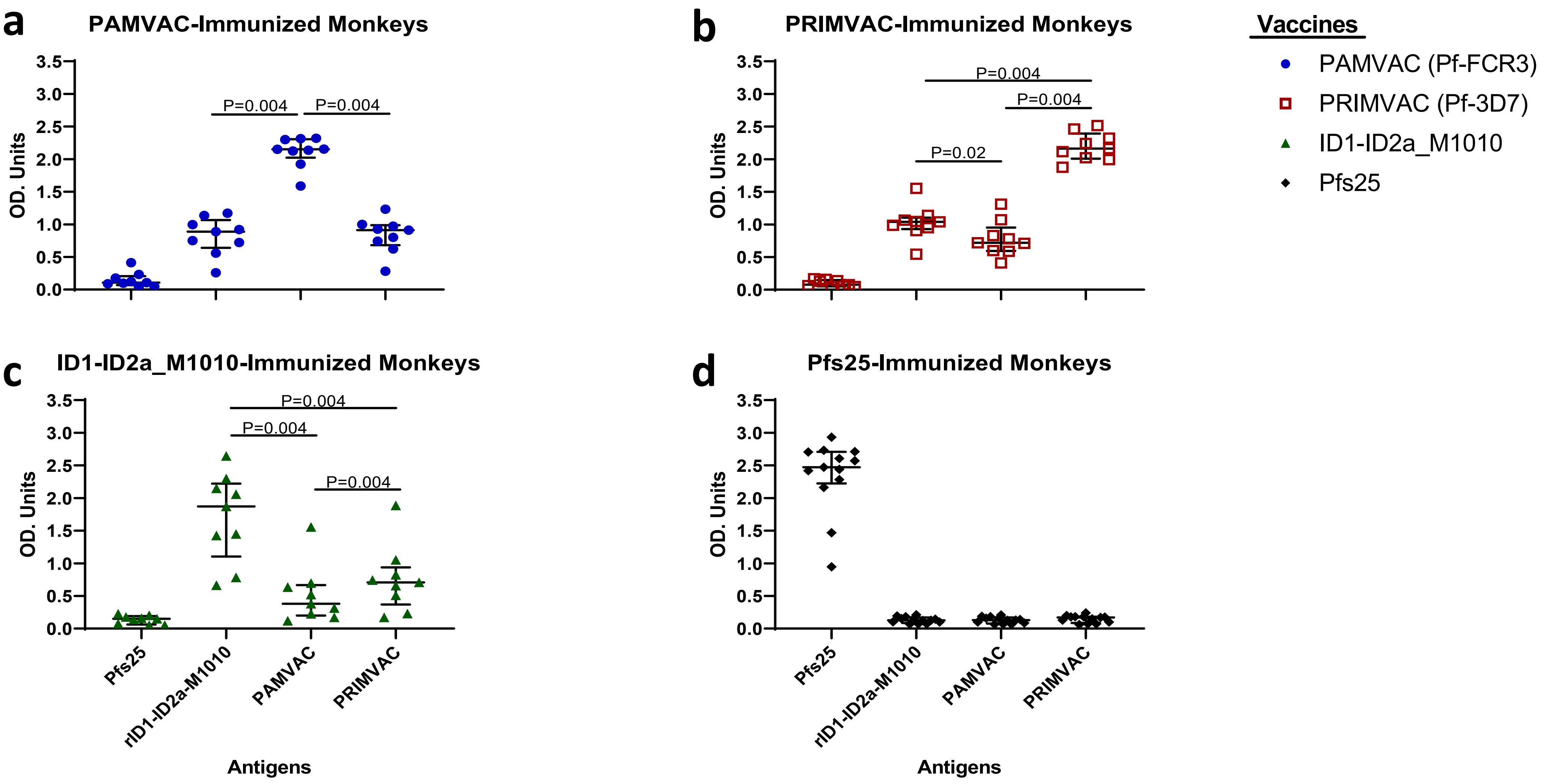

The cross-reactivity of vaccine-induced IgG by each antigen (**a**) PAMVAC (Pf-FCR3) (n = 9), (**b**) PRIMVAC (Pf-3D7) (n = 9), (**c**) rID1-ID2a-M1010 (n = 9) and (**d**) Pfs25 (n = 13) against was evaluated. For each group of vaccinated monkeys, the geometric mean and 95% CI of the antibody activity measured by ELISA and reported as optical density (OD) are shown. Mann-Whitney test was used to compare activity of vaccine-induced antibody to all the antigens and p-values are provided for PMV antigens when <0.05.

Supplementary Fig. 3: Heterologous and non-specific relationship between the ELISA titers of vaccine-induced antibodies and the surface reactivity and binding inhibitory activity at D70 post vaccination.

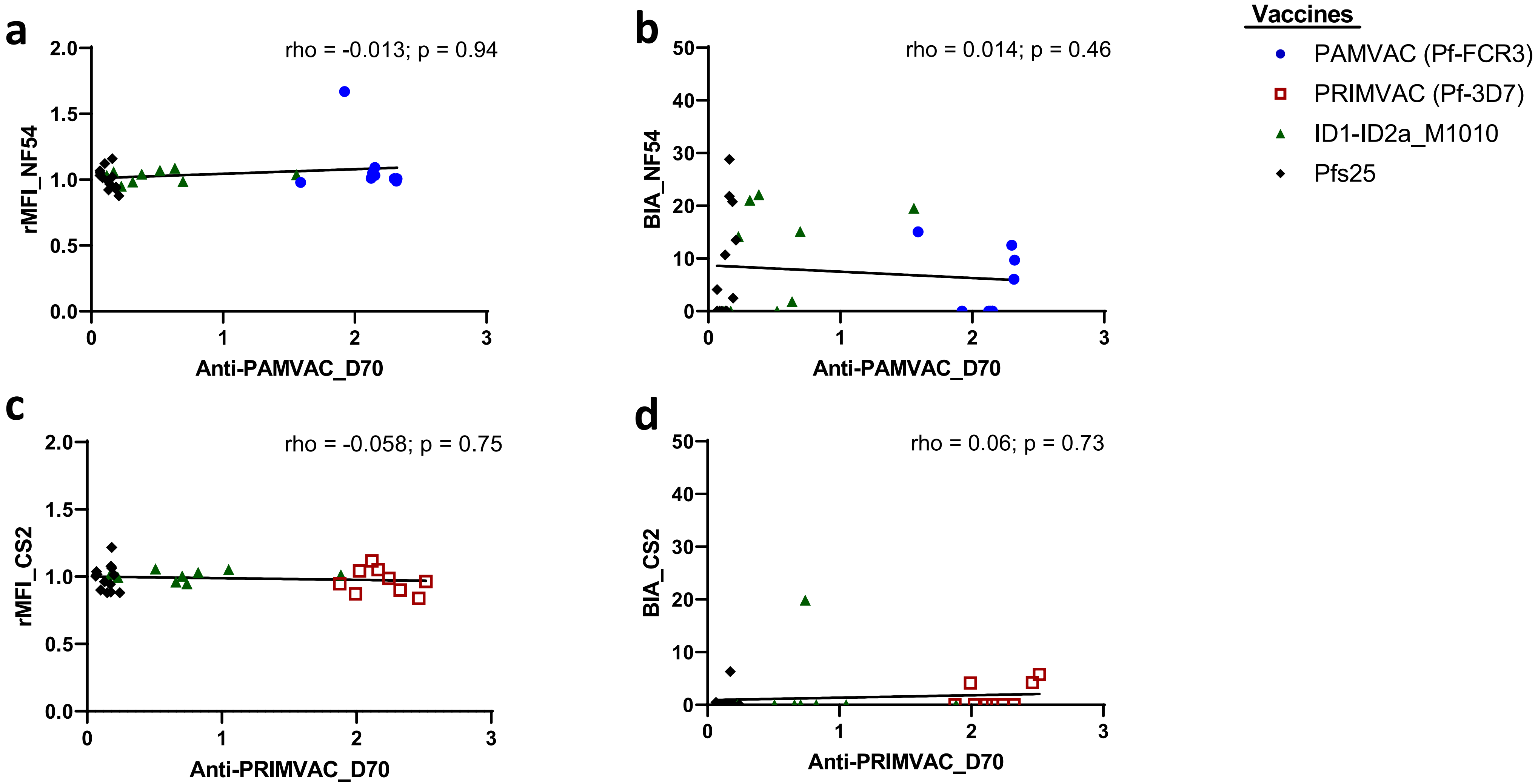

Spearman (rho) coefficients and *P* values are reported to describe the relationship between ELISA reactivity to PAMVAC antigen at D70 post vaccination and the surface reactivity (**a**) or binding inhibition activity (**b**) against NF54 parasite. The correlation between (**c**) surface reactivity and (**d**) BIA of antibodies to PRIMVAC) against CS2 parasite and presented.

Supplementary Fig. 4: Difference in time from D70 post vaccination 1 to CS2 infection

a

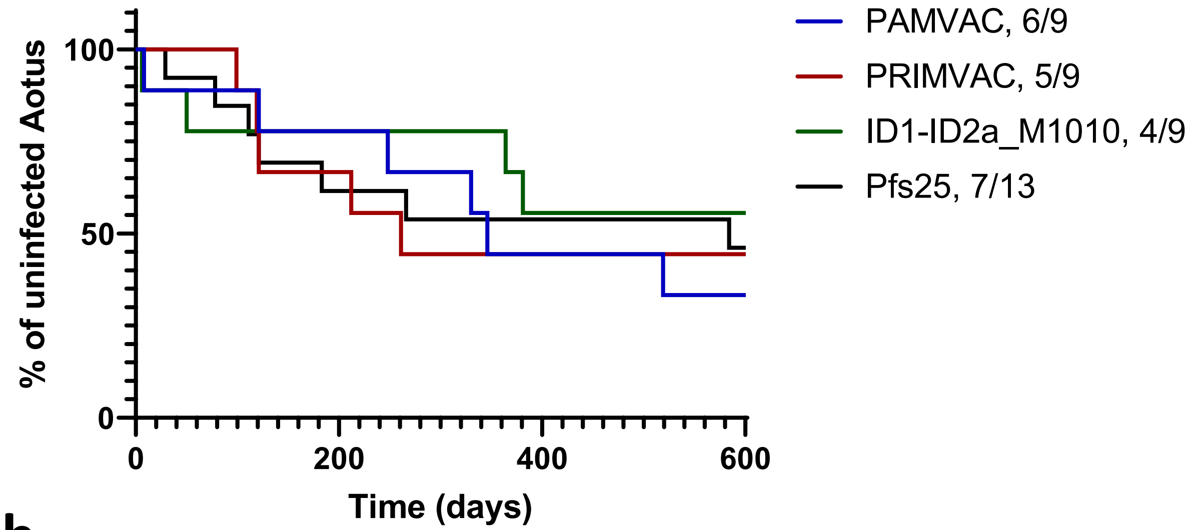

b

Comparison of vaccine groups (x vs. y)	Medians (x / y)	P-value
ID1-ID2a_M1010 vs. PAMVAC	207 / 289	0.91
ID1-ID2a_M1010 vs. Pfs25	207 / 121	0.93
ID1-ID2a_M1010 vs. PRIMVAC	207 / 121	1
PAMVAC vs. Pfs25	289 / 121	0.47
PAMVAC vs. PRIMVAC	289 / 121	0.27
Pfs25 vs. PRIMVAC	121 / 121	0.93

(**a**) Survival curves representing time to CS2 inoculation after D70 post vaccination for all the immunized monkeys are shown with the number of infected pregnant monkeys indicated for each group. **b,** Medians and P values comparing the time to CS2 inoculation in each vaccination group are provided to highlight difference was not significant.

Supplementary Fig. 5: Difference in PMV antibody levels from D70 post-vaccination 1 to the day of CS2 inoculation

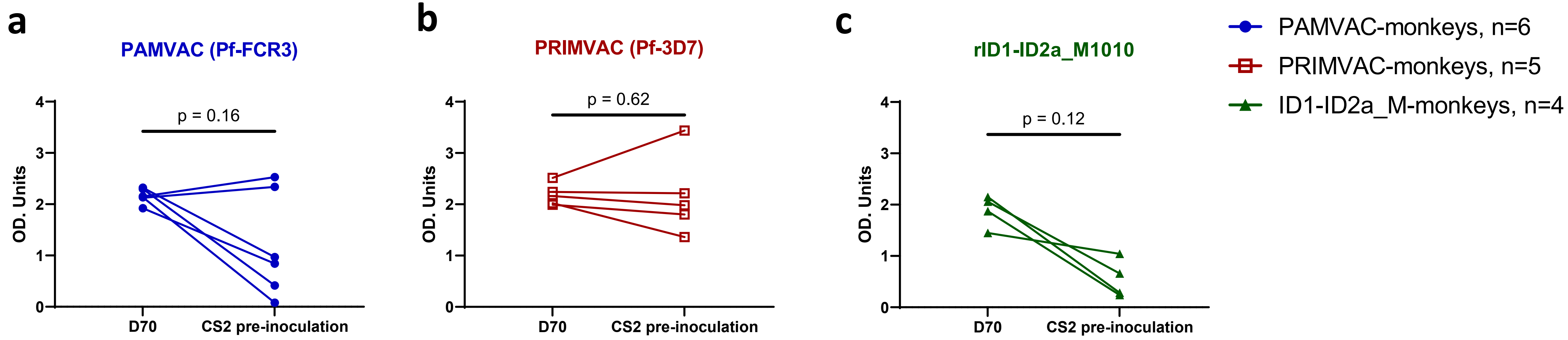

Change in ELISA titers 2 weeks after the last dose in the primary vaccine series (D70) compared to those measured at the time of CS2 parasite inoculation in individual monkey from (**a**) PAMVAC (n = 6), (**b**) PRIMVAC (n = 5) and (**c**) ID1-ID2a_M1010 (n = 4) vaccine groups is shown. Wilcoxon matched-pairs signed rank test was used to evaluate antibody decline or increase activity at the time of CS2 inoculation during pregnancy are p values are provided.

Supplementary Fig. 6: Dynamics of VAR2CSA antibodies following a CS2 infection during pregnancy in individual animal

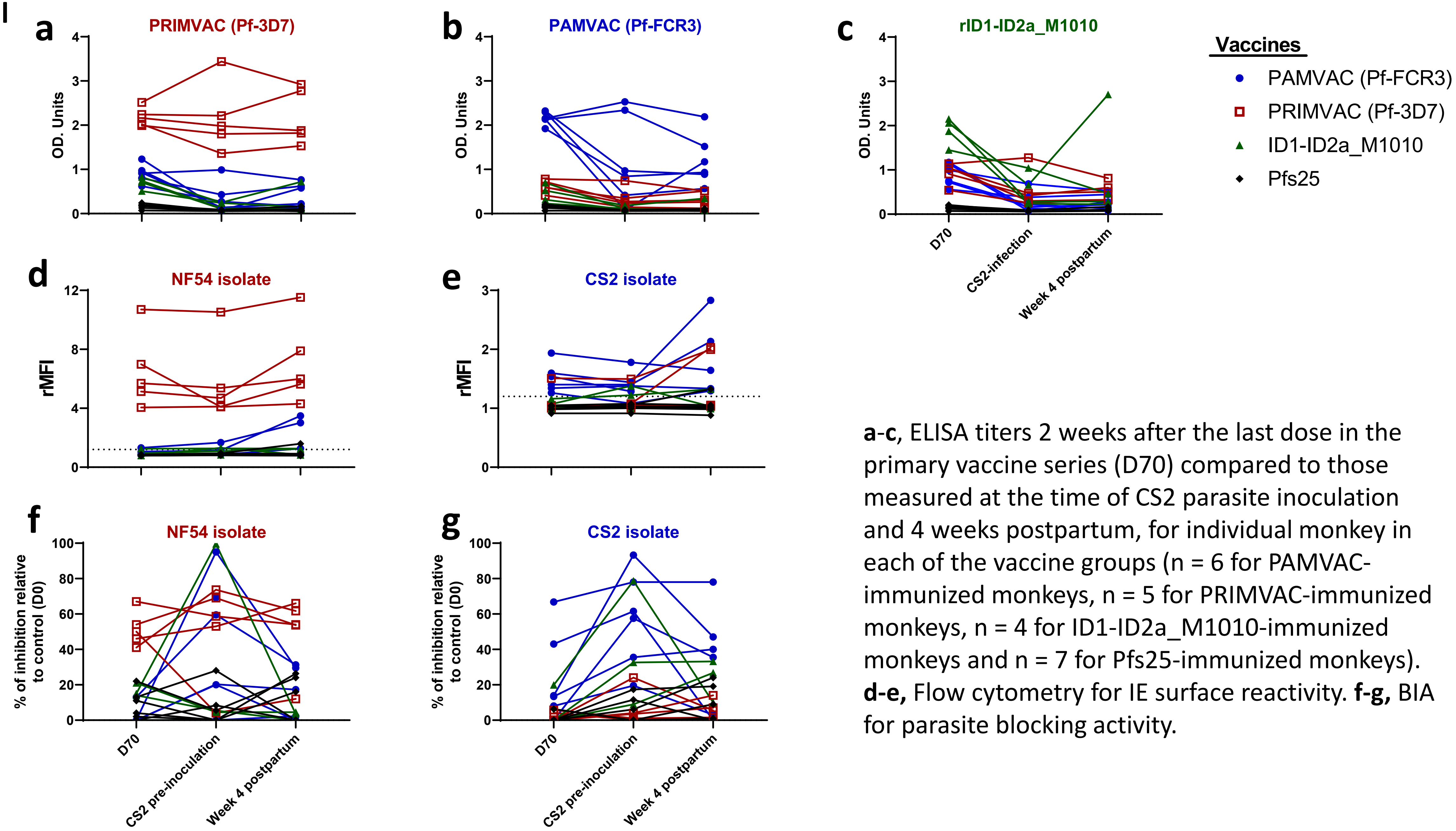

a-**c**, ELISA titers 2 weeks after the last dose in the primary vaccine series (D70) compared to those measured at the time of CS2 parasite inoculation and 4 weeks postpartum, for individual monkey in each of the vaccine groups (n = 6 for PAMVAC-immunized monkeys, n = 5 for PRIMVAC-immunized monkeys, n = 4 for ID1-ID2a_M1010-immunized monkeys and n = 7 for Pfs25-immunized monkeys). **d-e,** Flow cytometry for IE surface reactivity. **f-g,** BIA for parasite blocking activity.

Supplementary Fig. 7: Correlations between ELISA titers, surface reactivity of vaccine-induced antibodies and the CSA binding inhibitory activity at CS2 pre-inoculation.

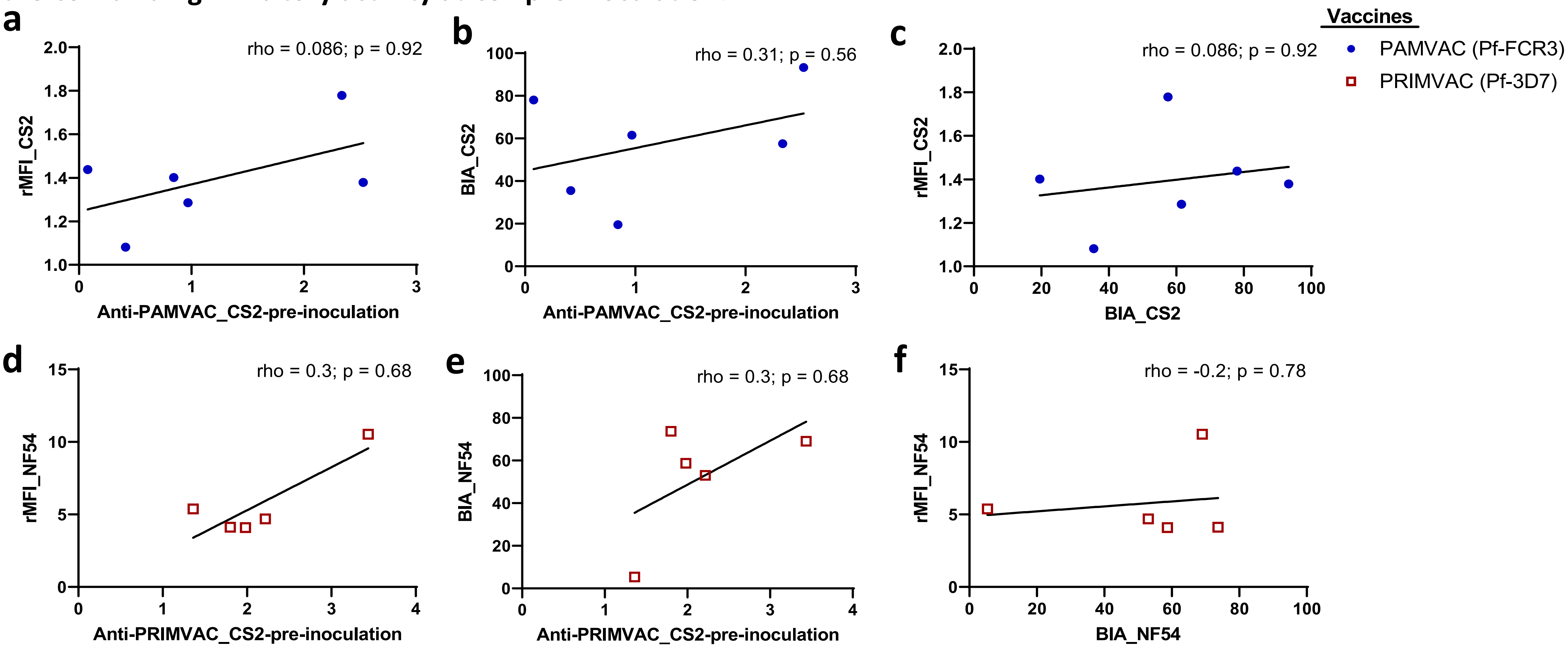

a-c, Spearman (rho) coefficients and *P* values are reported to describe the relationship between ELISA titers of PAMVAC antibodies in *Aotus* (n = 9) and surface reactivity (**a**) or binding inhibition activity (**b**) against CS2 parasite at the time of CS2 inoculation. The correlation between surface reactivity and BIA of PAMVAC antibodies is also shown (**c**). **d-f,** The same analyses were performed for PRIMVAC-induced antibodies in *Aotus* (n = 9) and presented. Data for animals immunized with PAMVAC (blue circle) and PRIMVAC (open red square) are shown.

Supplementary Fig. 8: Correlations between ELISA titers, surface reactivity of vaccine-induced antibodies and the CSA binding inhibitory activity at 4 weeks postpartum.

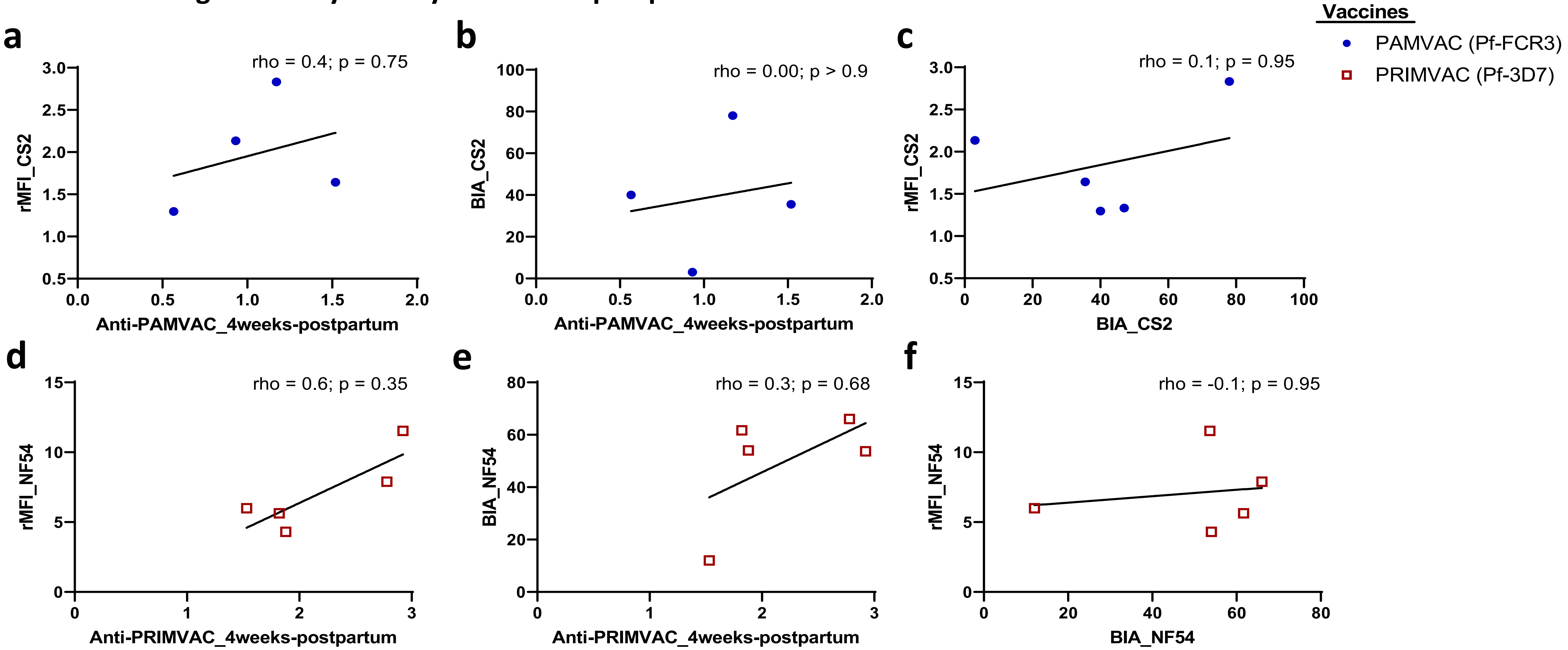

a-c, Spearman (rho) coefficients and *P* values are reported to describe the relationship between ELISA titers of PAMVAC antibodies in *Aotus* (n = 9) and surface reactivity (**a**) or binding inhibition activity (**b**) against CS2 parasite at 4 weeks postpartum. The correlation between surface reactivity and BIA of PAMVAC antibodies is also shown (**c**). **d-f,** The same analyses were performed for PRIMVAC-induced antibodies in *Aotus* (n = 9) and presented. Data for animals immunized with PAMVAC (blue circle) and PRIMVAC (open red square) are shown.

Supplementary Fig. 9: Correlations between ELISA titers, surface reactivity of vaccine-induced antibodies and the CSA binding inhibitory activity at CS2 pre-inoculation.

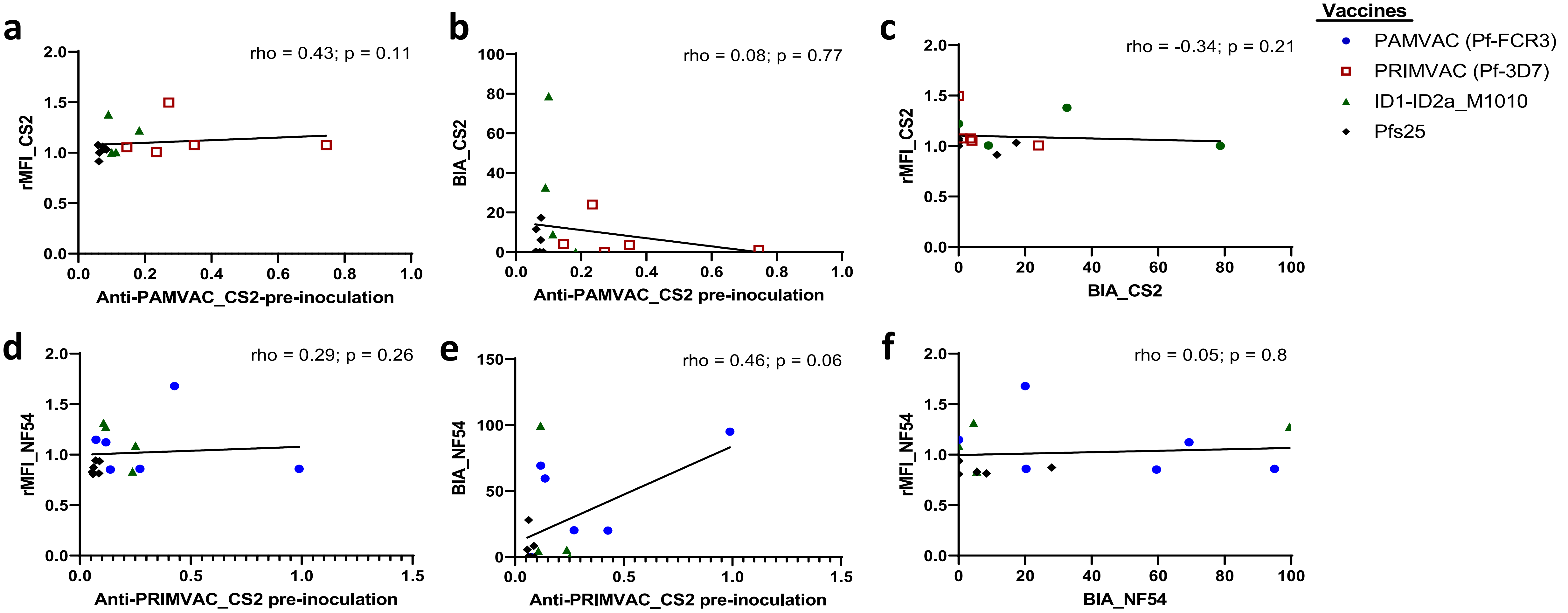

a-c, Spearman (rho) coefficients and *P* values are reported to describe the relationship between ELISA titers of heterologous antibodies to PAMVAC antigen in PRIMVAC-, ID1-ID2a_M1010- and Pfs25-immunized monkeys and surface reactivity (**a**) or binding inhibition activity (**b**) against CS2 parasite at CS2 pre-inoculation. The correlation between surface reactivity and BIA of PAMVAC reactive antibodies is also shown (**c**). **d-f,** The same analyses were performed for heterologous anti-PRIMVAC reactive antibodies and presented.

Supplementary Fig. 10: Correlations between ELISA titers, surface reactivity of vaccine-induced antibodies and the CSA binding inhibitory activity at 4 weeks postpartum.

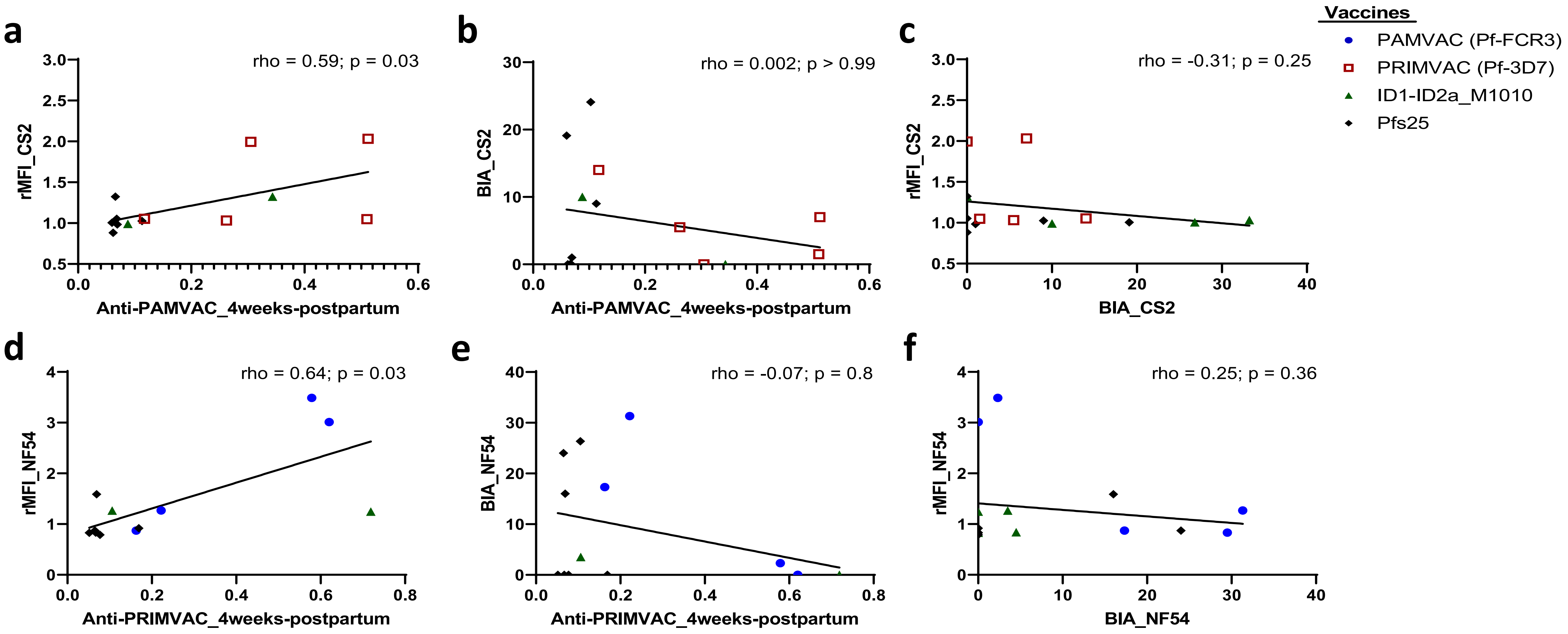

a-c, Spearman (rho) coefficients and *P* values are reported to describe the relationship between ELISA titers of heterologous antibodies to PAMVAC antigen in PRIMVAC-, ID1-ID2a_M1010- and Pfs25-immunized monkeys and surface reactivity (**a**) or binding inhibition activity (**b**) against CS2 parasite at CS2 pre-inoculation. The correlation between surface reactivity and BIA of PAMVAC reactive antibodies is also shown (**c**). **d-f,** The same analyses were performed for heterologous anti-PRIMVAC reactive antibodies and presented.

Supplementary Fig. 11: ELISA titer of PMV-induced antibodies in monkeys at D70, before CLP- or EPA-conjugated antigen boost, D14 and D56 post- boost.

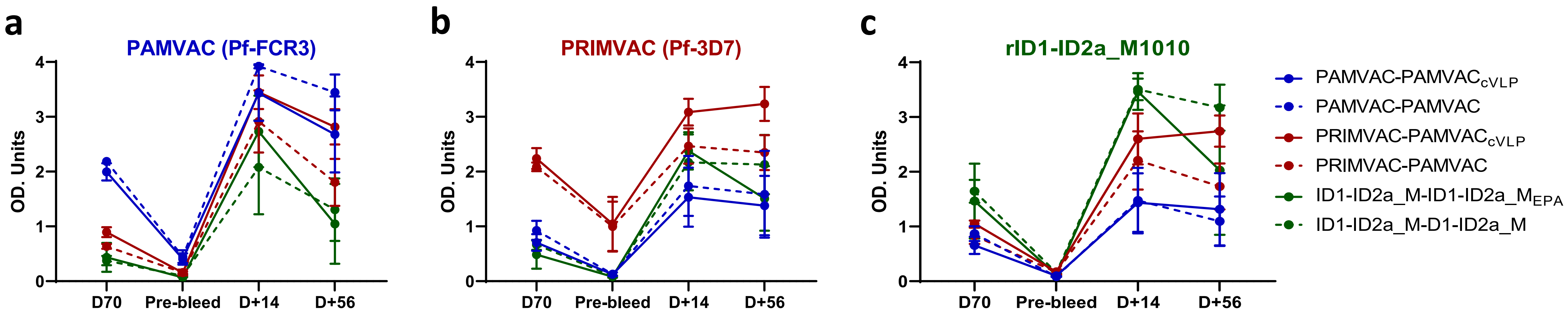

Activities of PMV-induced antibodies in *Aotus* monkeys following re-vaccination with monomer or nanoparticle immunogens. **a-c,** ELISA titer of IgG against PAMVAC (Pf-FCR3) (**a**), PRIMVAC (Pf-3D7) (**b**) and ID1-ID2a-M1010 (**c**) antigens were assessed in monkeys that received the nanoparticle antigens (solid-line, $PAMVAC_{cVLP}$ or ID1-ID2a-M1010-EPA) and those who received the monomer antigens (dashed-line). Samples collected at D70 post vaccination, before vaccination (Pre-bleed) and those collected at D14 and D56 post booster dose were analyzed.

Supplementary Fig. 12: Peripheral and Placental Parasitemia detected by thin blood smears

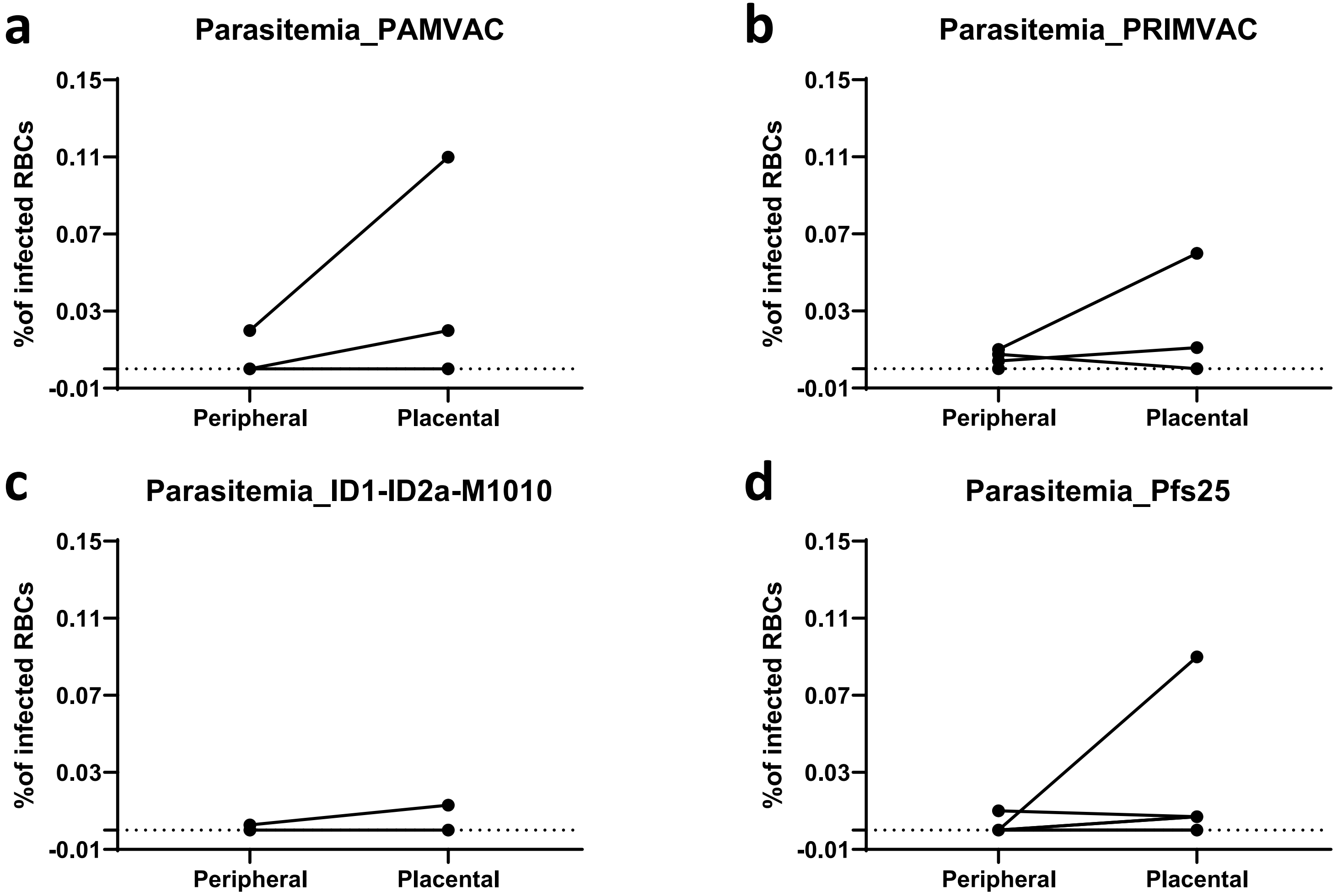

Blood smear slides from paired peripheral and placental blood samples collected- at C-section in monkeys immunized with PAMVAC (**a**), PRIMVAC (**b**), ID1-ID2a-M1010 (**c**) and Pfs25 (**d**) were examined by microscope. Parasitemia values are reported as percent of infected red blood cells (RBCs) per 15000 RBCs.

Supplementary Fig. 13: Age variation per vaccine group

a

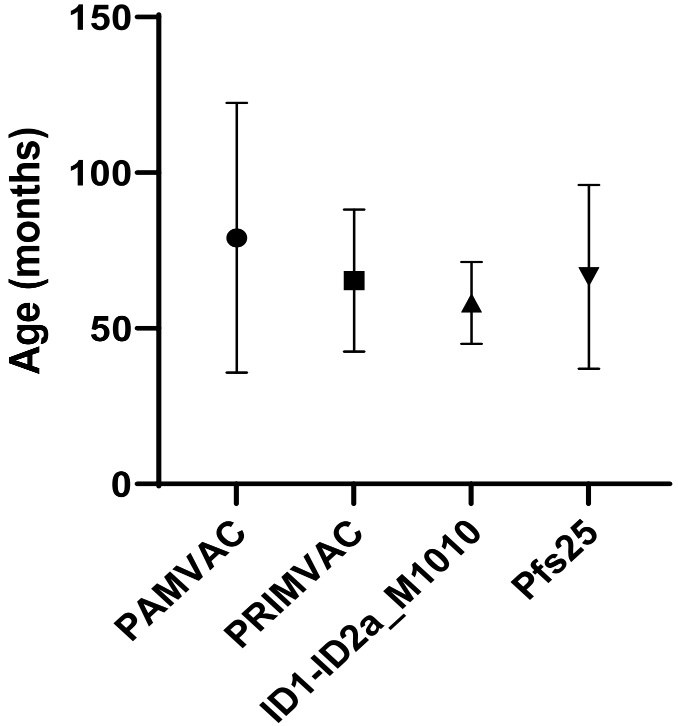

b

Mann-Whitney test

	PAMVAC	PRIMVAC	ID1-ID2a_M1010	Pfs25
**PAMVAC**				
**PRIMVAC**	0.47			
**ID1-ID2a_M1010**	0.5	0.74		
**Pfs25**	0.61	0.88	0.68	

a, Data representing the mean age with standard deviation (error bars) of the monkeys are provided for each vaccination group. **b**, The difference in age of the monkeys was compared among the different vaccine groups using a Mann-Whitney test are reported as table.

Supplementary Fig. 14: Biochemical and biophysical characterizations of recombinant ID1-ID2a_M1010 protein for identity, integrity and purity.

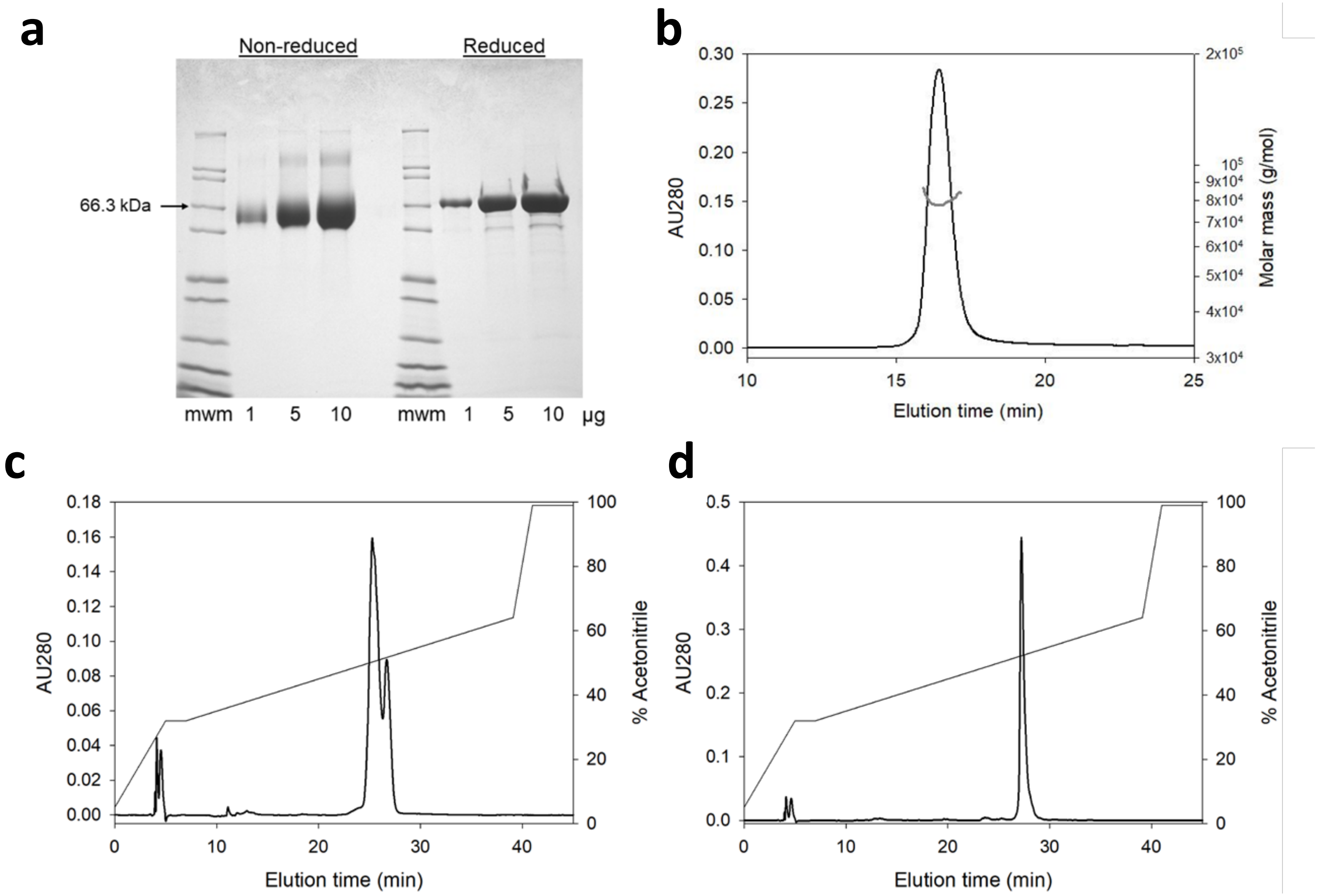

Characterization of purified BvID1-ID2a. **a**, shows BvID1-ID2a analyzed by Coomassie blue-stained SDS-PAGE under nonreduced and reduced conditions. **b**, shows an analytical size exclusion chromatogram with in-line multi-angle light scattering (SEC-MALS). **c**-**d**, shows reversed-phase HPLC analysis under non-oxidized conditions and reduced conditions, respectively.

Supplementary Table 1: Binding characteristic for lots of CS2 inoculums

	Number of Vials	Binding phenotype (CD36/CSA) *
PfCS2 Lot-2	29	38/06
PfCS2 Lot-3	26	49/02

Note: * Average bound infected red blood cells from 20 fields of 100X microscope, Binding receptors: CD36 and CSA

Supplementary Table 2: Vaccine treatment groups and formulations

a

Groups	Number of monkeys	Vaccine Formulation	*Pf* variant	Dose (µg/0.5 mL)	Alhydrogel (Aluminium content)
1	9	PAMVAC	FCR3	50	0.85mg
2	9	PRIMVAC	3D7	50	0.85mg
3	9	ID1-ID2a-M1010	M1010	50	0.404 mg
4	13	Pfs25	3D7	50	0.404 mg

b

Groups	Formulation Vac 1-3	Group # for Dose 4	Formulation Dose 4	Number of monkeys	Antigen Dose (µg/0.5 mL)	Aluminum Dose (µg/0.5 mL)
**1**	PAMVAC	**1A**	PAMVAC Monomer	3	50	850
		**1B**	PAMVAC VLP	4	12.5	850
**2**	PRIMVAC (3D7)	**2A**	PAMVAC Monomer	3	50	850
		**2B**	PAMVAC VLP	3	12.5	850
**3**	ID1-ID2a-M1010	**3A**	ID1-ID2a-M1010	2	50	404
		**3B**	ID1-ID2a-M1010-EPA	3	50	404

a, Formulation of the vaccines for the primary series of vaccination. *Pf = Plasmodium falciparum.* **b**, Booster dose vaccine formulation with antigen content per volume of 0.5 mL indicated.
